# Epidemiology of SARS-CoV-2 Infection Evaluated by Immunochromatographic Rapid Testing for the Determination of IgM and IgG Against SARS-CoV-2 in a Cohort of Mask Wearing Workers in the Metal-Mechanical Sector in an Area With a High Incidence of COVID-19

**DOI:** 10.3389/fpubh.2021.628098

**Published:** 2021-06-25

**Authors:** Susanna Esposito, Cosimo Neglia, Paola Affanni, Maria Eugenia Colucci, Alberto Argentiero, Licia Veronesi, Giulia Messina, Michela Deolmi, Nicola Principi, the Crown Study Group

**Affiliations:** ^1^University of Parma, Parma, Italy; ^2^University of Milan, Milan, Italy

**Keywords:** COVID-19, epidemiology, occupational medicine, SARS-CoV-2, public health

## Abstract

**Background:** Although the diagnosis of new coronavirus 2019 (COVID-19) is made through the identification of severe acute respiratory syndrome coronavirus 2 (SARS-CoV-2) in respiratory secretions by means of molecular methods, a more accurate estimation of SARS-CoV-2 circulation can be obtained by seroprevalence studies. The main aim of this study was to evaluate the true epidemiology of SARS-CoV-2 infection among workers in the metal-mechanical sector who never stopped working during the pandemic period in an area with a high incidence of COVID-19 and to define whether and how they could continue the work without appreciable risks during a second wave.

**Methods:** A total of 815 metal-mechanical workers who had never stopped working even during the pandemic period in three different factories in the Emilia-Romagna Region, Italy, and who had always used face masks during working hours, underwent a capillary blood rapid test for the determination of IgM and IgG against SARS-CoV-2 (COVID-19 IgG/IgM Rapid test, PrimaLab, Modena, Italy). In the event of a positive test, a nasopharyngeal was performed and tested for the presence of SARS-CoV-2.

**Results:** The detection of serum IgG/IgM against SARS-CoV-2 was significantly more common among workers employed in Parma (21/345, 6.1%) than among those employed in Calerno (7/242, 2.9%) or in Spilamberto (3/228, 1.3%) (*p* <0.001). The analysis of the role of the different variables as predictors of seropositivity for IgG/IgM against SARS-CoV-2 revealed that the presence of specific antibodies was strictly associated with a previous history of COVID-19-like symptoms (odds ratio [OR] 3.95, 95% confidence interval [CI] 1.9–8.2) and household members with COVID-19-like symptoms (OR 2.20, 95% CI 1.04–4.82).

**Conclusion:** This study shows that seropositivity to SARS-CoV-2 is low even among employees who did not interrupt their work during the lockdown phase in a region with a high incidence of COVID-19. The use of face masks appears effective in the avoidance of the transmission of SARS-CoV-2 in factories even in the presence of asymptomatic or mildly symptomatic workers, suggesting that work activities can continue if adequate infection control measures are used during a second wave.

## Background

A definitive diagnosis of new coronavirus 2019 (COVID-19) is made through the identification of severe acute respiratory syndrome coronavirus 2 (SARS-CoV-2) in the respiratory secretions of patients with suspected cases by means of molecular methods (1, 2). Generally, respiratory swabs are performed in symptomatic subjects, close contacts of patients with laboratory-confirmed cases and those coming from epidemic countries. However, as most infections remain asymptomatic or cause only mild symptoms and the sensitivity and specificity of laboratory tests can be suboptimal, the true epidemiology of SARS-CoV-2 is not precisely defined through respiratory swab analysis (3). A more accurate estimation of SARS-CoV-2 circulation and epidemiology can be obtained only by seroprevalence studies. When validated laboratory assays are used, the detection of specific IgM and IgG against SARS-CoV-2 can be considered a reliable measure of the real circulation of the virus and the number of infected people (4). Moreover, when studies are carried out in workplaces, such as factories, where it can be difficult to follow recommendations for infection prevention, including social distancing, the evaluation of seroprevalence can allow us to assess the real level of worker exposure and to implement the most effective preventive measures to reduce the risk of infection transmission. When a positive subject is detected, all the people who are in close contact with him/her can be carefully screened in order to avoid further spread of the virus and new infections. Moreover, given that the chance of reinfection in the short term is low if the serologic test is positive and the test for SARS-CoV-2 detection in the respiratory secretions is negative, positive workers can go back to work with a very low risk of transmission to others (5). In this study, a serological survey was performed with a validated assay for IgM and IgG antibodies against SARS-CoV-2 and carried out in a factory with three sections each located in a different province of Emilia-Romagna, a region of northern Italy with high SARS-CoV-2 circulation. The main aim was to evaluate the true epidemiology of SARS-CoV-2 infection among workers in the metal-mechanical sector who never stopped working during the whole epidemic period and to define whether and how they could continue their work without appreciable risks during a second wave.

## Methods

The project was carried out according to recommendations of the Emilia-Romagna Region and was approved by the Ethics Committee of Area Vasta Emilia Romagna Nord. In June 2020, upon the acceptance of participation by means of signing the informed consent form, which was explained in detail by staff of the Department of Medicine and Surgery of the University of Parma, the employees of the Crown Imballaggi company were enrolled in the study. All of them were metal-mechanical workers who had never stopped working even during the pandemic period. In the factories of the company, sited in the province of Reggio Emilia (Calerno), Parma (Parma), and Modena (Spilamberto) the use of face masks has been always mandatory during working hours.

Demographic data and essential information useful for reconstructing the medical history were collected. In particular, information relating to the period after January 1, 2020, was collected by means of a questionnaire administered at the time the participant signed the informed consent form to verify any contacts with subjects with SARS-CoV-2 infection, the presence of possible symptoms attributable to an infection and the use of personal protective equipment.

All participants underwent a capillary blood rapid test for the determination of IgM and IgG antibodies against SARS-CoV-2 (COVID-19 IgG/IgM Rapid test, PrimaLab, Modena, Italy). The test was an immunochromatographic test with specificity and sensitivity for IgM and IgG detection of 96.0 and 85.0 and of 98.0 and 100%, respectively that was validated and approved by the Emilia-Romagna Region, Italy (6). In the event of a positive test, a nasopharyngeal swab (eNAT, COPAN Diagnostics Inc., Brescia, Italy) was performed and tested for SARS-CoV-2 at the Hygiene and Public Health Laboratory of the Department of Medicine and Surgery of the University of Parma, Parma, Italy, as previously described (7). The rapid-test-positive workers were given a letter from the attending physician so that they could stop working pending the result of the swab test, which was available within 24 h of collection. In the event of a negative swab, workers could return to work immediately. If the serology was positive for anti-SARS-CoV-2 IgM and the swab was negative, work could be resumed only with a second negative swab. In the event of a positive swab, the Prevention Department of the city of residence of the worker was informed and took charge of the case.

Statistical data analyses were performed with Stata Release 12 (StataCorp, College Station, TX, USA). The sample was divided into groups (i.e., by sex, company office, and rapid positive-negative IgM-IgG test results) to perform a descriptive analysis. Quantitative variables (age) are reported as means ± standard deviation (SDs) and dichotomous variables (sex, yes or no answer) are reported as frequencies and percentages. Ages were compared across groups with the Wilcoxon-Mann-Whitney nonparametric test. The frequencies of the questionnaire responses and the dichotomous variables were compared with the chi-squared test in cases of frequencies >5. For exact frequencies ≤ 5, the Fisher's exact test was used. A univariate and multivariate logistic regression analysis was also conducted to calculate the probability ratios for yes or no answers to some questions.

## Results

During the study period, a total of 815 metal workers were enrolled (>90% of the employees in each factory). A small number of workers refused to participate in the study mainly because of concerns on secrecy and confidentiality of the data collected in the research. Table 1 shows their general characteristics according to the factory where they were working. Approximately one-quarter of the workers enrolled in the study suffered from COVID-19-like symptoms in the 5 months before the study period when the virus was circulating in the Emilia-Romagna Region, although only a few of them underwent a swab for SARS-CoV-2, and very few (three workers in Parma and one in Calerno) were hospitalized for COVID-19. Some of them had family members with COVID-19 or with COVID-19-like symptoms, and few of them were vaccinated against influenza. However, no significant difference was observed among the employees of the different factories.

**Table 1:** Epidemiological data of metal-mechanical workers working in an area with a high incidence of COVID-19, stratified by factory site.

	**Calerno** **(*N* = 242)**	**Parma** **(*N* = 345)**	**Spilamberto** **(*N* = 228)**	***p*-value**
Age, years	43.0 ± 12.2	44.8 ± 11.6	43.0 ± 10.1	0.46
Sex				0.27
Males, *n* (%)	167 (69.0)	258 (74.8)	169 (74.1)	
Females, *n* (%)	75 (31.0)	87 (25.2)	59 (25.9)	
Did you undergo a swab after January 1 for suspected COVID-19? Yes (%)	2 (0.8)	11 (3.2)	3 (1.3)	0.09
Was your swab positive?	1 (0.4)	2 (0.6)	0 (–)	
Have you had symptoms such as fever, cough, difficult breathing, cold, or changes in taste or smell since January 1st? Yes (%)	56 (23.1)	100 (29.0)	65 (28.5)	0.25
Have you had a diagnosed case of COVID-19 (positive swab) in your family (household)? Yes (%)	5 (2.1)	12 (3.5)	4 (1.8)	0.36
If no, have any of your family members had COVID-19-like symptoms? Yes (%)	48 (9.8)	78 (2.6)	41 (18.0)	0.47
Did you receive a flu vaccination?	18 (0.4)	33 (9.6)	30 (13.2)	0.11

The detection of serum IgG/IgM against SARS-CoV-2 was significantly more common among workers employed in Parma (21/345, 6.1%; 19 with IgG only and two with IgM only) than among those employed in Calerno (7/242, 2.9%; five with IgG only and two with IgM only) or in Spilamberto (3/228, 1.3%; two with IgG and one with IgM) (*p* < 0.001). Nasopharyngeal swabs carried out in all 31 seropositive subjects detected SARS-CoV-2 in only two workers, both in Parma in subjects positive for IgG only who were totally asymptomatic. Table 2 summarizes the characteristics of metal-mechanical workers with serology positive or negative for SARS-CoV-2. In comparison with the seronegative individuals, seropositive individuals were significantly older, had previously underwent a swab for COVID-19-like symptoms significantly more often and reported COVID-19-like symptoms for themselves and their household members significantly more frequently. Table 3 shows the univariate and multivariate logistic regression analysis of the associations between epidemiological data and the dependent variables of seropositivity for IgG/IgM against SARS-CoV-2. The presence of specific antibodies was strictly associated with a previous history of COVID-19-like symptoms: this result was confirmed in the univariate as well as multivariate logistic regression analysis.

**Table 2:** Epidemiological data of metal-mechanical workers working in an area with a high incidence of COVID-19 stratified by serological immunochromatographic rapid test results.

	**Negative** **(*N* = 783)**	**Positive (*N* = 31)**	***p*-value**
Age, years	43.5 ± 11.4	51.0 ± 9.4	<0.001
Sex			0.51
Males, *n* (%)	573 (73.1)	21 (67.7)	
Females, *n* (%)	211 (26.9)	10 (32.3)	
Did you undergo a swab after January 1 for suspected COVID-19? Yes (%)	10 (1.3)	6 (19.5)	<0.001
Have you had symptoms such as fever, cough, difficult breathing, cold, or changes in taste or smell since January 1st? Yes (%)	203 (25.9)	18 (58.1)	<0.001
Have you had a diagnosed case of COVID-19 (positive swab) in your family (household)? Yes (%)	21 (2.7)	– (–)	0.37
If not, have any of your family members had COVID-19-like symptoms? Yes (%)	156 (19.9)	11 (35.5)	<0.05
Did you receive a flu vaccination?	75 (9.6)	6 (19.3)	0.077

**Table 3:** Univariate and multivariate logistic regression analysis showing associations between epidemiological data and the dependent variables of seropositivity for IgG/IgM against SARS-CoV-2.

	**Univariate**		**Multivariate**	
**Variable**	**OR (95% CI)**	***p*-value**	**OR (95% CI)**	***p*-value**
Sex	0.77 [0.36–1.67] Intercept: 0.04 [0.02-0.06]	0.513 <0.001	0.98 [0.43–2.21]	0.96
Age group				
<30	–	–	–	
30–40	4.06 [0.46–35.31]	0.203	3.70 [0.41–32.71]	0.24
40–50	0.47 [0.02–7.58]	0.595	0.47 [0.03–7.67]	0.60
50–60	11.48 [1.52–86.27]	<0.05	12.15 [1.60–92.49]	<0.05
>60	5.48 [0.48–62.07]	0.17	6.06 [0.50–72.52]	0.15
Intercept age group	0.01 [0.01–0.06]	<0.001	–	–
Previous history of COVID-19-like symptoms	3.95 [1.9–8.2] Intercept: 0.02 [0.01–0.03]	<0.001 <0.001	3.89 [1.70–8.91]	<0.01
Flu vaccination	2.26 [0.90–5.70] Intercept: 0.03 [0.01–0.05]	0.08 <0.001	1.57 [0.58–4.28]	0.37
Household members with COVID-19-like symptoms	2.20 [1.04–4.82] Intercept: 0.03 [0.01–0.04]	<0.05 <0.001	1.38 [0.57–3.30]	0.472
Intercept multivariate	–	–	0.004 [0.001–0.03]	<0.001

*CI, confidence interval; OR, odds ratio*.

## Discussion

Emilia-Romagna was one of the Italian regions with the greatest COVID-19 incidence and the highest numbers of disease-related deaths. In the whole region, in which live more than 4 million inhabitants, on June 9, 2020, the day after the end of the study period, a total of 27,946 laboratory-confirmed cases of SARS-CoV-2 had been diagnosed, and 4,185 COVID-19-related deaths had been ascertained. However, the results of this study seem to indicate that, despite COVID-19 being a relevant medical problem in the region, only a marginal part of the population has been exposed to the virus and had developed an immune response against SARS-CoV-2 in the 1st months from pandemic onset. This conclusion is not doubted by the lack of evaluation of the IgA response in the studied subjects as the improvement given by IgA detection for identification of seropositive individuals is, if present, too small to invalid study conclusions (8). On the contrary, results are strongly supported by the observation that it seems highly unlikely that a relevant number of infected subjects had not developed an immune response or that had lost anti SARS-CoV-2 specific antibodies in a short time after infection. Slight differences among the studied factories were evidenced without statistical significance. This reflects the differences in COVID-19 incidence among the provinces of the region where the total number of diagnosed cases was slightly higher in Parma and Reggio Emilia than in Modena (9). In this regard, these findings are quite in agreement with those previously reported in other countries worldwide (10). If the presence of antibodies could be, at least in part, associated with immunity, these results seem to indicate that the pandemic was far from over and that the achievement of herd immunity would take a long time. The lowest calculated disease-induced herd immunity level for SARS-CoV-2 infection was approximately 43% (11), a value several times higher than the seropositivity rate found in this study and even higher than the percentage of seropositivity reported in populations exposed to the virus without effective preventive measures, including lockdown (12).

Moreover, this study confirms that most of the individuals with SARS-CoV-2 infection are asymptomatic or poorly symptomatic (13, 14). Very few seropositive metal-mechanical workers enrolled in this study had signs or symptoms of disease severe enough to require hospitalization, and although more than 27% of them had clinical manifestations that could suggest COVID-19, only 7.2% of these underwent a nasopharyngeal swab for the confirmation of the diagnosis. Attempts to diagnose COVID-19 through nasopharyngeal swab testing were more common in Parma than in other towns, probably because in Parma, a greater number of close contacts or household members of enrolled metal-mechanical workers had had a diagnosed case of COVID-19. However, the data collected with this study highlight how nasopharyngeal swab testing represents the fundamental cornerstone for the early diagnosis of COVID-19 and, in the pandemic period, should be performed as soon as symptoms suggestive of disease appear in any subject or in any family member to avoid the spread of SARS-CoV-2. The evidence that seropositivity, i.e., the demonstration of exposure to the virus, was strictly related to both the history of symptoms potentially associated with COVID-19 and the development of COVID-19-like symptoms among household members strongly supports this conclusion.

Regarding the risk of the spread of infection among the metal-mechanical workers enrolled in this study, this seems very low, although results of the study could slightly underestimate this risk as some of the workers remained absent from the fabric for some days during the study period due to disease other than COVID-19 or personal reasons. However, only 2 out of 815 (0.2%) studied individuals had a laboratory-confirmed SARS-CoV-2 infection in the absence of symptoms at enrolment. On the other hand, all of them strictly followed SARS-CoV-2 infection control measures, including the use of face masks during working hours, and these measures permit the avoidance of viral transmission during working hours (15, 16). As it has been reported that a non-marginal proportion of asymptomatic adults can persistently shed the virus (17, 18), it seems recommendable that in factories such as those included in this study, activity can continue even in the case of a second wave, but all the measures for infection control, including the use of face masks, must be carefully followed, and nasopharyngeal swab testing should be performed in individuals with signs and symptoms suggestive of COVID-19.

## Conclusion

This study shows that seropositivity to SARS-CoV-2 is low even among employees who did not interrupt their work during the lockdown phase in a region with a high incidence of COVID-19. The use of face masks appears effective in the avoidance of the transmission of SARS-CoV-2 in factories even in the presence of asymptomatic or poorly symptomatic workers, suggesting that work activities can continue in the presence of adequate infection control measures during a second wave. Education on infection control measures and the use of personal safety devices against respiratory pathogens appears mandatory and should be promptly implemented in different workplaces. In addition, a prompt diagnosis with nasopharyngeal swab testing at the territorial level is extremely important in individuals with COVID-19-like symptoms or close contacts with infected people to implement appropriate management and the isolation of individuals with positive cases.

## Data Availability Statement

The original contributions presented in the study are included in the article/supplementary material, further inquiries can be directed to the corresponding author/s.

## Ethics Statement

The studies involving human participants were reviewed and approved by Ethics Committee of Emilia-Romagna Area Vasta Nord. The patients/participants provided their written informed consent to participate in this study.

## Crown Working Group

Neglia Cosimo, Argentiero Alberto, Abate Luciana, Antoniol Giulia, Autore Giovanni, Bernardi Luca, Bertolini Davide, Bossù Gianluca, Cannata Giulia, Caporilli Chiara, Caramia Mara, Chinè Vincenzo, Chiopris Giulia, Ciavola Lorenzo, Conte Cristiano, Cremonini Isabella, Cusenza Francesca, D'Alvano Tiziana, Davino Giusy, Deolmi Michela, Di Sario Riccardo, Falcinella Francesca, Fanelli Umberto, Fracchiolla Adriana, Gagliardi Martina, Gianní Giuliana, Gnocchi Margherita, Grassi Federica, Guidarini Marta, Labate Marialuisa, Lattanzi Claudia, Laudisio Serena, Marinelli Francesca, Mariotti Elena, Massa Serena, Meoli Aniello, Messina Giulia, Monaco Sara, Motta Matteo, Nicoletti Laura, Orlando Serena, Paglialonga Letizia, Pappalardo Marco, Passadore Lucrezia, Pecora Francesco, Persico Federica, Procaccianti Michela, Ratti Chiara, Santoro Angelica, Scavone Sara, Sodini Chiara, Sogni Francesco and Torelli Lisa.

## Author Contributions

SE proposed the project, supervised the enrolment, and wrote the first draft of the manuscript. CN and AA were the study coordinators of the project. PA, MC, and LV performed the laboratory analyses. GM and MD performed data entry and participated in enrolment. NP co-wrote the manuscript, provided a scientific contribution, and critically revised the manuscript. All the authors have read and approved the final manuscript.

## Conflict of Interest

The authors declare that the research was conducted in the absence of any commercial or financial relationships that could be construed as a potential conflict of interest.
